# A *Trypanosoma cruzi* Genome Tandem Repetitive Satellite DNA Sequence as a Molecular Marker for a LAMP Assay for Diagnosing Chagas' Disease

**DOI:** 10.1155/2020/8074314

**Published:** 2020-02-22

**Authors:** Diego Ordóñez, Pedro Fernández-Soto, Ana M. Fernández-Martín, Beatriz Crego-Vicente, Begoña Febrer-Sendra, Juan García-Bernalt Diego, Belén Vicente, Julio López-Abán, Moncef Belhassen-García, Antonio Muro, Manuel A. Patarroyo

**Affiliations:** ^1^Animal Science Faculty, Universidad de Ciencias Aplicadas y Ambientales (U.D.C.A), 111166 Bogotá, Colombia; ^2^Infectious and Tropical Diseases Research Group (e-INTRO), Biomedical Research Institute of Salamanca, Research Centre for Tropical Diseases at the University of Salamanca (IBSAL-CIETUS), Faculty of Pharmacy, University of Salamanca, 37007 Salamanca, Spain; ^3^School of Medicine and Health Sciences, Universidad del Rosario, 112111 Bogotá, Colombia; ^4^Fundación Instituto de Inmunología de Colombia (FIDIC), 111321 Bogotá, Colombia

## Abstract

Chagas' disease is a neglected tropical disease caused by *Trypanosoma cruzi* which is endemic throughout Latin America and is spread by worldwide migration. Diagnosis is currently limited to serological and molecular techniques having variations regarding their sensitivity and specificity. This work was aimed at developing a new sensitive, applicable, and cost-effective molecular diagnosis technique for loop-mediated isothermal amplification-based detection of *T. cruzi* (Tc-LAMP). The results led to determining a highly homologous satellite repeat region (231 bp) among parasite strains as a molecular marker for diagnosing the disease. Tc-LAMP was performed correctly for detecting parasite DNA (5 fg for the CL Brener strain and 50 fg for the DM28, TcVI, and TcI strains). Assay results proved negative for DNA from 16 helminth species and 7 protozoa, including *Leishmania* spp. Tc-LAMP based on the highly repeated *T. cruzi* satellite region is thus proposed as an important alternative for diagnosing *T. cruzi* infection, overcoming other methods' limitations such as their analytic capability, speed, and requiring specialized equipment or highly trained personnel. Tc-LAMP could be easily adapted for point-of-care testing in areas having limited resources.

## 1. Introduction

American trypanosomiasis, or Chagas' disease, is a zoonotic disease, usually consisting of chronic parasitic infection caused by the kinetoplastid protozoan *Trypanosoma cruzi*. The World Health Organization (WHO) recognizes Chagas' disease as one of the 20 neglected tropical diseases (NTD) [1] and one of the 13 most NTD worldwide [2]. Chagas' disease was considered a strictly rural disease for many decades; however, socioeconomic changes, rural exodus, deforestation, and urbanization have transformed the disease's epidemiological profile, making it an increasingly urban phenomenon and a major public health problem [3]. The disease can currently be found in 21 Latin American countries, and it has been estimated that at least 8 million people are infected worldwide. Migration has increased the disease's incidence, and it has been spread to other continents [2, 4].

Chagas' disease diagnosis depends on the phase in which a patient is found to be. Parasitemia is high during the acute phase and the congenital form, as well as in reactivations caused by immunosuppression during the chronic phase. The parasite can be detected by microscopy in peripheral blood by thin or thick smear with Giemsa staining [5]. Sensitivity can be increased by concentration techniques such as microhematocrite concentration or Strout (double centrifugation) methods [6]. Techniques like hemoculture or xenodiagnosis have already been abandoned [7]. The parasite can sometimes be detected in cerebrospinal liquid [8]. Direct parasitological methods usually prove negative in 30%-60% of patients during the disease's chronic phase (having minimum parasitemia). Diagnosis is thus serological during this phase, based on detecting anti-*T. cruzi* IgG antibodies.

Conventional serological techniques (indirect immunofluorescence (IFI), indirect hemagglutination (IHA), and multienzyme assays (ELISA)) use the complete parasite or antigen mixtures as an antigen. However, less conventional serological assays use purified and/or recombinant antigens and synthetic peptides. Such techniques are more sensitive than direct observation methods but involve problems regarding poor specificity associated with antigen differences between recombinant proteins from different parasite lineages [9]. As no available serological test has 100% sensitivity and specificity, the WHO defines the diagnosis of the disease during its chronic phase by positivity in two serological tests carried out using different methods. A third test must be performed in case of discrepancy between the forgoing two results to confirm or discard infection [8]. Discrepancies are often due to crossed reactions with other trypanosomatids, including *Leishmania* spp.

Detecting *T. cruzi* DNA in peripheral blood by polymerase chain reaction (PCR) has become increasingly more used during the last few years, and the Special Program for Research and Training in Tropical Diseases (TDR-WHO) supported a comparative international study of PCR for detecting *T. cruzi* [10]. Kinetoplastid DNA, satellite DNA repeated sequences, and ribosomal RNA genes are the most used amplification targets [11]. PCR has proven useful during acute-phase or chronic-phase reactivations due to its greater sensitivity compared to microscopy methods [12].

PCR use during the chronic phase is debatable because it gives a positive result in 40%-70% of patients who have previously been diagnosed by conventional serological methods, depending on the degree of parasitemia, sample volume, DNA purification, PCR target region, the study population's characteristics, and the great genetic variability between the parasite's discrete typing units (DTUs). Furthermore, a negative result does not exclude infection [13].

PCR has also been used for following up treatment efficacy so that a positive result at the end of treatment would indicate therapeutic failure [14]. Real-time (quantitative) PCR (qPCR) has been developed enabling parasite DNA detection and quantification from clinical samples, although having very variable levels regarding analytical reliability, specificity, and sensitivity [15–18] thereby hampering its standardization for use in routine clinical matters. Such methods are impracticable in endemic areas lacking resources since they are techniques requiring sophisticated equipment and qualified personnel and are expensive, making them unfeasible for use in field conditions in endemic areas having scarce resources.

Other sensitive and specific molecular techniques, which are simpler, faster, and cheaper than PCR and its variants, must thus be used in diagnosing Chagas' disease, such as nucleic acid isothermal amplification, i.e., loop-mediated isothermal amplification (LAMP). Such technique has recently been revealed as an alternative having great potential for diagnosis in endemic areas [19–22]. Progress has been described in recent years regarding new LAMP methodologies for Chagas' disease diagnosis [19]. However, such laboratory tools are still being developed and larger amounts of reagents and materials are needed which could increase the value of diagnosis in communities living in endemic areas, mainly in the third world. This work has thus been aimed at developing a new sensitive, applicable, and cost-effective LAMP assay for the molecular detection of *T. cruzi*.

## 2. Methods

### 2.1. DNA from *T. cruzi* and Other Parasites

A DNeasy Blood & Tissue Kit (Qiagen, Hiden, Germany) was used for DNA extraction according to the manufacturer's instructions. The *T. cruzi* genomic DNA (gDNA) used in this study was obtained from CL Brener and Dm28 in vitro parasite strain culture maintained at the University of Granada's Biochemistry and Molecular Parasitology Department, Spain. A NanoDrop spectrophotometer (ND-1000) was used for measuring DNA from both strains which was subsequently diluted with ultrapure distilled water to final 0.5 ng/*μ*L concentration. Serial 10-fold dilutions (1 × 10^−1^ to 1 × 10^−9^ ng/*μ*L) from both *T. cruzi* DNA strains were then prepared and stored at -20°C until use. The prepared DNA was used as a positive control in all LAMP reactions for evaluating the molecular assays' analytical sensitivity and specificity.

Twenty-three DNA samples taken from several other parasites available in our laboratory were also evaluated for determining LAMP assay specificity, including trematodes (*Schistosoma mansoni*, *S. haematobium*, *Fasciola hepatica*, *Amphimerus* sp., *Dicrocoelium dendriticum*, and *Echinostoma caproni*), cestodes (*Hymenolepis diminuta*, *Echinococcus granulosus*, *Taenia saginata*, and *T. solium*), nematodes (*Anisakis simplex*, *Brugia pahangi*, *Loa*, *Mansonella perstans*, *Strongyloides venezuelensis*, and *Ascaris suum*), and protozoa (*Giardia intestinalis*, *Cryptosporidium parvum*, *Entamoeba histolytica*, *Plasmodium malariae*, *P. vivax*, *Leishmania infantum*, and *L. donovani*). All DNA sample concentrations were measured with a NanoDrop spectrophotometer (ND-1000) and then diluted with ultrapure water to a final 0.5 ng/*μ*L concentration and kept at -20°C until use.

### 2.2. Designing LAMP Primers

The GenBank database (http://www.ncbi.nlm.nih.gov/) was searched for identifying possible sequences which could have been proven useful for detecting *T. cruzi* DNA. Once the sequences for starting the analysis had been located, they were saved in FASTA text-based format for handling and edition using BioEdit Sequence Alignment Editor v7.2.5 [23]. It was then ascertained whether the sequences were also available in the TriTrypDB database (http://tritrypdb.org/tritrypdb/) which stores the complete *T. cruzi* genome. The sequences were then compared using the basic local alignment search tool (BLAST; https://blast.ncbi.nlm.nih.gov/Blast.cgi) for ascertaining the degree of homology and/or difference from the other specific trypanosomatid sequences available in the database. The resulting sequence was used for designing specific primers for *T. cruzi* amplification using LAMP Designer software (http://www.premierbiosoft.com/isothermal/lamp.html). Thermo Fisher Scientific synthesized the selected primers.

### 2.3. LAMP Assay for *T. cruzi*

LAMP reaction mixtures (25.2 *μ*L) each contained 40 pmol FIP and BIP primers, 5 pmol F3 and B3 primers, 5 pmol LB and LF primers, 2.5 mM dNTPs (Intron), 0.8 mM MgSO_4_ (New England Biolabs, UK), 1 M betaine (Sigma, USA), 1x isothermal amplification buffer-20 mM Tris-HCl (pH 8.8), and 8 U Bst polymerase 2.0 WarmStart (New England Biolabs, UK) with 2 *μ*L (1 ng) of template DNA.

LAMP reactions were performed in 0.5 mL microcentrifuge tubes incubated in a heating block (K Dry Bath) at 63°C-65°C for 30 min, 45 min, and 60 min to optimize the reaction; the temperature was then increased to 80°C for 5-10 min to deactivate the enzyme and stop the reaction. DNA contamination was prevented by using sterile tools at all times, each analysis step is performed in separate work areas, and reaction tube manipulation is minimized. Template DNA was replaced by ultrapure water as a negative control in each LAMP reaction. *T. cruzi* gDNA (10-fold serial dilutions as mentioned above) was also amplified for determining the LAMP assay's lower detection limit. LAMP assay specificity for only amplifying *T. cruzi* DNA was tested against 23 DNA samples from other parasites.

### 2.4. Detecting LAMP-*T. cruzi* Products

The LAMP amplification results were visualized by adding 2 *μ*L of 1 : 10 diluted SYBR Green I fluorescent dye (Invitrogen, Carlsbad, California, USA) to the reaction tubes. Green fluorescence was observed in positive LAMP reactions whilst keeping its original orange in negative reactions. LAMP products (3-5 *μ*L) were also monitored by electrophoresis in 1.5-2% agarose gel electrophoresis to corroborate colorimetric results. The gels were visualized under UV light and photographed using an ultraviolet Gel Documentation System (Uvitec, UK).

## 3. Results

### 3.1. Searching for and Selecting Molecular Targets for Designing LAMP Primers

Table 1 summarizes eight molecular targets found in literature and database analysis that have been traditionally used in PCR studies for *T. cruzi* DNA or RNA amplification.

The TriTrypDB database (http://tritrypdb.org) was used for a BLASTN local search and alignment analysis [32] to verify these target sequences in the *T. cruzi* genome; different degrees of homology were obtained when comparing them to different parasite strains (Table 2).

The satellite nuclear DNA 195 bp sequence [24] was selected as a target for LAMP primer design following in silico analysis of the selected sequences and studying their main characteristics using TriTrypDB. This small 195 bp sequence was included in a larger 938 bp one (Tcruzi_30851; TriTrypDB), identical for many strains and highly repeated (up to 9%) in the *T. cruzi* genome. Furthermore, PCR amplification of parasite DNA analytical sensitivity and specificity has been proven in a number of studies. This small 195 bp sequence was manually extended to 231 bp to guarantee producing LAMP primers regarding the minimum size supported by LAMP Designer software. A single set of six primers (F3, B3, FIP, BIP, LF, and LB) was produced.  Table 3 shows the characteristics of the primers and nucleotide sequences.

### 3.2. Setting Up the LAMP Assay for *T. cruzi*: Tc-LAMP

**Figure 1:**
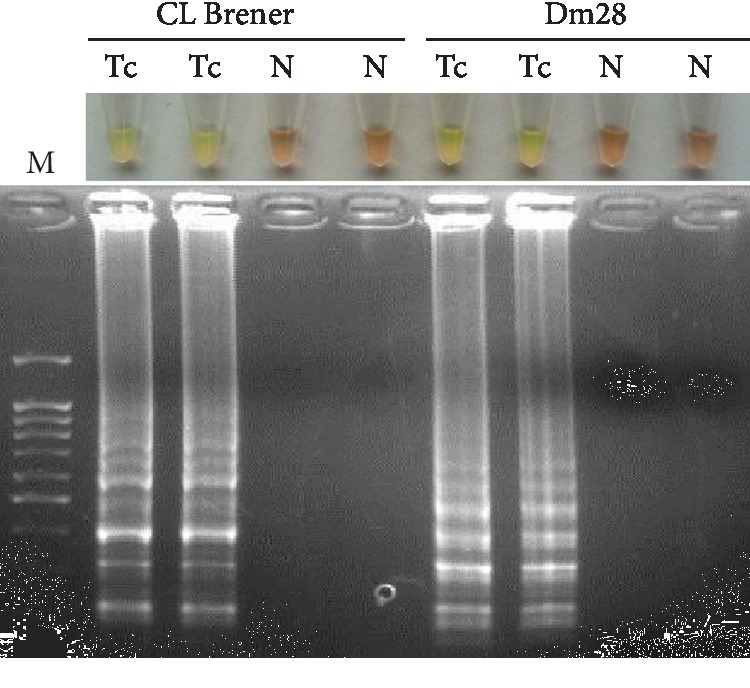
LAMP detection of *Trypanosoma cruzi* CL Brener and Dm28 strain DNA. Lanes Tc: *T. cruzi* DNA (0.5 ng/*μ*L) from CL Brener and Dm28 strains; lanes N: negative controls (ultrapure water, no DNA). M: molecular weight marker (100 bp Plus Blue DNA Ladder).

The best amplification results and test reproducibility for CL Brener and Dm28 strains were obtained when the reaction mixtures were incubated at 65°C for 60 min. Color changes were clearly observed when adding SYBR Green I to the postamplification tubes, as was a ladder-like pattern in agarose gel electrophoresis (Figure 1).

**Figure 2:**
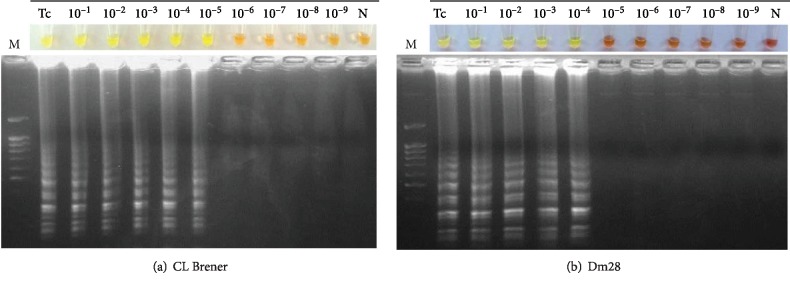
Assessment of Tc-LAMP analytical sensitivity for *Trypanosoma cruzi* using CL Brener and Dm28 strain gDNA serial dilutions. (a) Tc-LAMP sensitivity for the CL Brener strain. (b) Tc-LAMP sensitivity for the Dm28 strain. Lanes Tc: *T. cruzi* CL Brener and Dm28 strain gDNA (0.5 ng/*μ*L); lanes 10^−1^-10^−9^: 10-fold serial dilutions; lanes M: molecular weight marker (100 bp Plus Blue DNA Ladder).

The CL Brener strain DNA amplification detection limit was 5 fg when evaluating Tc-LAMP reaction's analytical sensitivity, whereas the Dm28 strain detection limit was 50 fg (Figure 2).

**Figure 3:**
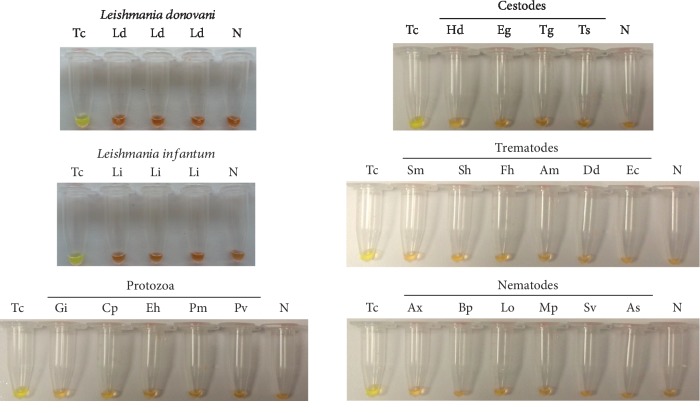
Tc-LAMP specificity: visual examination of LAMP products by adding SYBR Green I. Lanes Tc: *T. cruzi* CL Brener or Dm28 strain DNA (0.5 ng/*μ*L); lanes N: negative controls (ultrapure water, no DNA). Trematode DNA in lanes Sm (*Schistosoma mansoni*), Sh (*S. haematobium*), Fh (*Fasciola hepatica*), Am (*Amphimerus* sp.), Dd (*Dicrocoelium dendriticum*), and Ec (*Echinostoma caproni*). Nematode DNA in lanes Ax (*Anisakis simplex*), Bp (*Brugia pahangi*), Lo (*Loa loa*), Mp (*Mansonella perstans*), Sv (*Strongyloides venezuelensis*), and As (*Ascaris suum*). Cestode DNA in lanes Hd (*Hymenolepis diminuta*), Eg (*Echinococcus granulosus*), Tg (*Taenia saginata*), and Ts (*T. solium*). Protozoan DNA in lanes Gi (*Giardia intestinalis*), Cp (*Cryptosporidium parvum*), Eh (*Entamoeba histolytica*), Pm (*Plasmodium malariae*), and Pv (*P. vivax*). Leishmania donovani DNA in lane Ld (*Leishmania donovani* in triplicate). Leishmania infantum DNA in lane Li (*Leishmania infantum* in triplicate).

Tc-LAMP assay specificity was also tested using heterogeneous DNA samples as controls. Positive amplification was only observed using *T. cruzi* DNA (CL Brener or Dm28). DNA samples from other specimens tested were not amplified, thereby indicating that cross-reactions had not occurred. Colorimetric assay positive and negative results were clearly visualized and no ladders for bands were observed on agarose gels (Figure 3).

## 4. Discussion

Chagas' disease is an endemic protozoosis on the American continent, affecting its poorest communities, and has become a public health problem in many Latin American countries. Early diagnosis is essential for effective treatment to eliminate the parasite. However, both direct parasitological and serological methods have problems regarding their sensitivity and specificity. Molecular methods represent an alternative to be used for amplifying *T. cruzi* DNA. This work has described a LAMP technology-based molecular method (Tc-LAMP) developed for detecting the parasite.

The available databases (i.e., TriTrypDB and GenBank) were used for selecting eight sequences from different *T. cruzi* genome regions for designing useful primers for the LAMP assay; these sequences had been previously used for detecting *T. cruzi* DNA and that of its variants by PCR [16, 26, 28]. A small 195 bp highly repeated (up to 9%) nuclear DNA sequence in the *T. cruzi* genome [24] was selected as the potential molecular target for amplification following in silico analysis. This sequence is identical for many strains and has 100% identity with the CL Brener and Tulahuen c12 strains. This sequence's drawback lies in its small size for primer design, since LAMP Designer software requires a minimum 200-300 bp for proper operation. The genome sequence included in a greater 938 bp region was located in the *T. cruzi* genome to solve this problem. The selected sequence could thus be manually expanded to 231 bp, nearly the software's minimum size. The analysis gave a single set of 6 primers which included two loop primers. A repetitive nuclear DNA sequence amplifying a 231 bp fragment was selected from these in silico studies and used for developing the LAMP technique for *T. cruzi* DNA amplification.

The reaction conditions were established in line with the previous work by our group for detecting DNA from other parasites [33, 34]. CL Brener strain gDNA (a strain first used in sequencing the *T. cruzi* genome) [35] was amplified along with that from the Dm28 strain (a strain sequenced in 2014 and used in biochemical, immunological, gene expression, cell biology, and new drug development studies) [36]. Parasites involved in the domestic cycle belong to the CL Brener strain whilst circulating parasites in the sylvatic cycle belong to the Dm28 strain.

The Tc-LAMP detection limit was different for both strains tested: 5 fg for CL Brener and 50 fg for Dm28. Such differences regarding analytical sensitivity were probably due to variations among the DTUs distributed into six groups (TcI-TcVI) according to their molecular differences. There were also differences regarding the amount of their repetitive sequence content and genomic size, varying extensively among strains, even in the same DTU [37]. Thus, CL Brener strain genomic size (TcVI) was estimated to be 152.12 Mb, whilst Dm28 strain genomic size (TcI) was estimated to be 111.40 Mb. Satellite DNA was more abundant in the CL Brener strain (giving greater analytical sensitivity in our test) than in the Dm28 strain. The specificity studies involved a battery of available gDNA extracted from other parasites. The results were negative for the 16 helminth gDNA and the seven protozoan cDNA analyzed. It is worth highlighting the lack of gDNA detection in *Leishmania* species (*Leishmania donovani* and *Leishmania infantum*), although future studies would involve testing many more *Leishmania* species since coinfection with *T. cruzi* is a frequently occurring problem, especially in certain areas of the American continent. Moreover, trypanosomatid genomes share a high level of synteny [38, 39].

Only two LAMP assays were available for detecting *T. cruzi* gDNA when our LAMP assay was originally developed (both developed by the same research group) [40, 41]. A specific LAMP was developed for detecting *T. cruzi*, *T. congolense*, *T. evansi*, and *T. brucei gambiense* in one of the studies, using 5.8S rRNA gene internal transcribed spacer sequences (ITS2) for *T. brucei gambiense*, 18S rRNA gene ITS2 for *T. congolense* and *T. cruzi*, and the VSG RoTat1.2 surface glycoprotein for *T. evansi* [40]. The *T. cruzi* detection limit was 1 fg using Tulahuen strain DNA (TcVI human strain), such limit being greater than that detected in our assay. However, although specificity was evaluated in that study using gDNA from African trypanosomes and other protozoa, such as *Toxoplasma*, *Theileria*, and *Babesia*, specificity against *Leishmania* spp. species and *T. rangeli* mainly present on the American continent was not evaluated [42].

Significant advances in using LAMP for Chagas' disease diagnosis have currently been described [19]. However, more reagents and materials are needed for this which could thus increase the value of diagnosis for communities living in endemic areas, mainly in the third world.

## 5. Conclusion

Our study has described developing a specific LAMP method (Tc-LAMP) based on detecting a highly repeated and homologous satellite region among different strains for the sensitive and specific detection of *T. cruzi* DNA. The results led to proposing this marker/Tc-LAMP method as an accurate tool for Chagas' disease diagnosis, highlighting its advantages regarding speed, not needing specialized laboratory equipment or the need for highly trained personnel for running such tests.

## Figures and Tables

**Table 1:** Main molecular targets for *Trypanosoma cruzi* DNA or RNA detection using PCR or RT-PCR. GenBank database accession numbers, the amplification method used (PCR for DNA or RT-PCR for RNA), base pair length, and references are indicated.

Molecular target	Accession number	PCR/RT-PCR	Base pairs	Reference
Nuclear satellite DNA	AY520036	PCR	195	[24]
Putative flagellar calcium-binding protein	Z54193	PCR	692	[25]
E13 repeated element	M74536	PCR	220	[26]
Kinetoplast minicircle variable region	AJ748042	PCR	330	[27]
24S ribosomal RNA alpha-subunit	L14468	RT-PCR	100	[28]
Ribosomal intergenic spacer	M63895	PCR	1174	[29]
Transposon-like E22 repeated element	X95485	PCR	863	[30]
Subtelomeric conserved junction	AF100651	PCR	822	[31]

**Table 2:** Similarity degree among TriTrypDB sequences.

Target	Identity	*E* value	Strain	Notes^∗^
Nuclear satellite DNA	100%	3*e* − 96	CL Brener/Tulahuen c12	Most abundant repetitive sequence in *T. cruzi* genome (up to 9%)
Putative flagellar calcium-binding protein	99%	0.0	CL Brener/JRc14/Tulahuen c12/Dm28c/Marinkellei	Sequence present in a single copy. Low sensitivity
E13 repeated element	97%	0.0	Tulahuen c12/CL Brener/Dm28c/Esmeraldo	Abundant repetitive sequence in *T. cruzi* genome (up to 7%)
Variable region of kinetoplast minicircles	100%	6*e* − 172	CL Brener/Tulahuen c12/JRc14/Esmeraldo	Variable region with low specificity for LAMP primer design
24S ribosomal RNA alpha-subunit	97%	2*e* − 44	JRc14/SylvioX10/Dm28c/Tulahuen c12/Marinkellei/CL Brener/Esmeraldo	Small size for LAMP design and very conserved in trypanosomatids
Ribosomal intergenic spacer	95%	2*e* − 107	JRc14/SylvioX10/Dm28c/Tulahuen c12/Esmeraldo/Brener	Scarcely used for *T. cruzi* detection
Transposon-like E22 repeated element	98%	0.0	CL Brener/Tulahuen c12/Esmeraldo/Dm28c	Scarcely used for *T. cruzi* detection
Subtelomeric conserved junction	96%	0.0	Tulahuen c12/JRc14/CL Brener/Esmeraldo/Dm28c	Scarcely used for *T. cruzi* detection

^∗^Consult the bibliography shown in Table 1 for more details about sequences.

**Table 3:** Sequences and characteristics of the primers used.

Primer	Len	Tm	3′dG	GC rate (%)	Sequence (5′ to 3′)
F3	18	60.8	-1	50	AACTATCCGCTGCTTGGA
B3	18	60.2	-0.4	50	AAGAGCTCGCGAAATTCC
FIP (F1c-F2)	41				CCCACCATTCACAATCGGAAACCACTCGGCTGATCGTTTT
BIP (B1c-B2)	41				AGTCAGAGGCACTCTCTGTCAACCAAGCAGCGGATAGTTC
F2	19	60.3	-0.7	50	CACTCGGCTGATCGTTTT
F1c	20	65.1	0.1	55.6	CCCACCATTCACAATCGGAAAC
B2	18	65.1	-0.9	50	CCAAGCAGCGGATAGTTC
B1c	22	72.5	-0.9	50	AGTCAGAGGCACTCTCTGTCAA
LF	19	60.1	0.1	50	TTGGACCACAACGTGTGAT
LB	20	64.4	-0.4	50	TTCACACACTGGACACCAAA

## Data Availability

The data used to support the findings of this study are available from the corresponding author upon request.
